# Characteristics of Dough Rheology and the Structural, Mechanical, and Sensory Properties of Sponge Cakes with Sweeteners

**DOI:** 10.3390/molecules26216638

**Published:** 2021-11-02

**Authors:** Agata Marzec, Jolanta Kowalska, Ewa Domian, Sabina Galus, Agnieszka Ciurzyńska, Hanna Kowalska

**Affiliations:** Department of Food Engineering and Process Management, Institute of Food Science, Warsaw University of Life Sciences-SGGW, 159c Nowoursynowska St., 02-776 Warsaw, Poland; jolanta_kowalska@sggw.edu.pl (J.K.); ewa_domian@sggw.edu.pl (E.D.); sabina_galus@sggw.edu.pl (S.G.); agnieszka_ciurzynska@sggw.edu.pl (A.C.); hanna_kowalska@sggw.edu.pl (H.K.)

**Keywords:** sponge cakes, erythritol, maltitol, trehalose, texture, color, microtomography

## Abstract

Changes in the rheological properties of dough, as well as the microstructural, mechanical, and sensory properties of sponge cakes, as a function of the substitution of sucrose in a formulation with maltitol, erythritol, and trehalose are described. Moreover, the relationship between the examined properties was investigated. The replacement of sucrose with maltitol or trehalose did not affect the consistency index, whereas erythritol caused a decrease in its value. X-ray tomography was used to obtain the 2D and 3D microstructures of sponge cakes. All studied sweeteners caused the sponge cakes to have a typical porous structure. Erythritol and maltitol resulted in about 50% of the pores being smaller than 0.019 mm^2^ and 50% of the pores being larger than 0.032 mm^2^. Trehalose resulted in a homogeneous microstructure, 98% of whose pores were similar in size (0.019 to 0.032 mm^2^). The sponge cakes with polyols had a higher structure index than did the trehalose and sucrose samples. There were also significant differences in color parameters (lightness and chromaticity). The crust of the sponge cake with sweeteners was lighter and had a less saturated color than the crust of the sponge cake with sucrose. The sponge cake with maltitol was the most similar to the sponge cake with sucrose, mainly due to the mechanical and sensory properties. Trehalose led to the samples having high adhesiveness, which may limit its application as a sucrose substitute in sponge cake. Sensory properties were strongly correlated to cohesiveness, adhesiveness, and springiness and did not correlate to the 2D and 3D microstructures. It was found that 100% replacement of sucrose allows for a porous structure to be obtained. These results confirm that it is not the structure, but most of all the flavor, that determines the sensory perception of the sponge cakes.

## 1. Introduction

The dough for sponge cakes is a complex emulsion and foam system. Sponge cakes are a baked product with a porous structure and classified as a foam-type cake. The foam structure is due to the content of eggs that form a stable emulsion [1,2,3] and the presence of air pores, introduced during mixing, that are separate and surrounded by a continuous phase [4]. Many authors have concluded that the viscosity and stability of pores are the most essential parameters for final quality, especially cake volume [5,6,7]. The final aerated structure and volume of sponge cakes depend on both the aeration of the dough and the expansion of bubbles during baking [8]. The rheological properties of the dough and the texture of the sponge cake are significantly affected by the ingredients, the dominant ones being protein and sugar [2,9,10,11]. The development of the dough structure depends on the flour used and, above all, on the content of starch and gluten [12]. Starch is responsible for the viscoelastic properties and determines the textural characteristics of the sponge cake. Gluten determines the flexibility and adhesion of the dough. Low availability of water and lipids causes starch granules to achieve only partial gelatinization and form a solid network of interconnected and swollen starch grains [13]. The rheological properties of the dough are complex and are viscoelastic systems showing the properties of multiphase liquids or solids [12]. Sucrose is the most frequently applied sweetener in the confectionery industry, and it is also applied as a texture-forming agent. Despite being classified as nonreducing, sucrose inverts to glucose and fructose at higher temperatures. Both these monosaccharides participate in Maillard reactions with amino acids to form the important flavor and browning compounds [9,14]. In cakes, sucrose delays starch gelatinization and protein denaturation [11].

Considering the high incidence of diabetes and obesity, it is a common practice to replace sucrose with sweeteners [10,14,15]. Trehalose is a natural disaccharide that is half as sweet as sucrose and possesses a low insulin response [11]. Trehalose has high thermal stability and is a nonreducing sugar able to form non-hygroscopic “glazes”. Although trehalose is widely used in frozen dough, few studies have considered its application in sponge cake. Trehalose has a positive effect on the extension of shelf life, the maintenance of texture and shape, and the modification of taste [16]. Research has shown that trehalose has beneficial effects on the treatment of impaired cognitive and learning abilities [17] and the prevention of neurodegeneration [18].

Some investigations were conducted to determine the effect of sugar replacement in cakes with polyols (hydrogenated carbohydrates) such as sorbitol, mannitol, lactitol, maltitol, and xylitol [19] as well as glycerol and erythritol [10,11]. The energy value of polyols is lower than that of sucrose. They decrease the water content in food products, while their high osmotic pressure enhances the preservation effect [20]. The viscosity of standard muffin dough was compared to dough containing polyols during heating and subsequent cooling, and it was found that the sugar alcohols had a similar influence on the dough setting to sucrose [21]. In addition, the delayed starch gelatinization temperature and increased viscosity during heating demonstrate the usability of polyols as sucrose replacements [11,21]. Compared with sugars, polyols (e.g., xylitol, maltitol, sorbitol, and erythritol) are poorly absorbed and provide fewer calories and a lower number of glycemic responses. These characteristics make them popular as sweeteners in diabetic and low-energy food [11,16].

Maltitol is an alcohol containing multiple hydroxyl groups and is capable of stabilizing the native structure of globular proteins and of reducing their denaturation [22]. Due to its low energy value and sweet taste, maltitol is increasingly being used in the food industry, including the confectionery industry [22,23]. Maltitol replaces sugar on a weight-by-weight basis and provides 2.40 kcal/g [11].

Erythritol, a four-carbon sugar alcohol, is safe even at high intake values [24]. It is non-caloric, non-cariogenic, and non-glycemic, and has antioxidative properties that fit well into the functional food concepts, to which it confers a healthy image [13,25]. According to the Regulation of 2008, such polyols as erythritol and maltitol may be added to confectionery products at the quantum satis level, but only to low-energy and sugar-free products. It is important for sponge cake makers to obtain products with the porosity, appearance, and composition that consumers demand. Growing interest should be taken in low-calorie foods, as well as foods with few ingredients, which may mitigate the stigma associated with the lack of healthfulness of processed food products.

This tendency results both from the health problems of the population and the growing care for the condition of the body. Erythritol affects glucose metabolism and reduces lipid peroxidation, thereby improving the damage caused by the oxidative stress involved in the pathogenesis of diabetes [25,26]. Sponge cakes can be made according to the traditional recipe, i.e., eggs, sugar, and flour, without any other additives. Therefore, they are used in some diets and can be given to infants.

Hence, our knowledge of the influence of individual ingredients on the quality of baking is essential when improving or developing a recipe. Although the use of erythritol and maltitol in cake is relatively well described in the literature, there remains a lack of knowledge about the use of trehalose [11]. Moreover, there is no information on the influence of polyols and trehalose on the sensory properties and 2D and 3D microstructures of sponge cakes. The structure of food foams influences their mechanical properties and sensory perception [1,27] as well as digestion and lipolysis, which contribute to nutrition and health [28].

To better understand the effect of the type of sweetener on texture and sensory perception, it is necessary to analyze the 3D microstructure of the sponge cake (in terms of porosity, pore size distribution, and structural thickness). X-ray microtomography (μCT) has been proven to constitute a noninvasive but reliable tool for food quality analysis [29].

Considering the above, the aim of this study was to determine the effect of sweeteners on the rheological properties, density, and pH value of dough and on the water content, water activity, density, color, 2D and 3D structure, mechanical properties, and sensory properties of sponge cakes. Correlations between structural, mechanical, and sensory indicators were analyzed using principal component analysis (PCA).

## 2. Results and Discussion

### 2.1. Rheological Properties, Density, and pH of Dough

**Figure 1:**
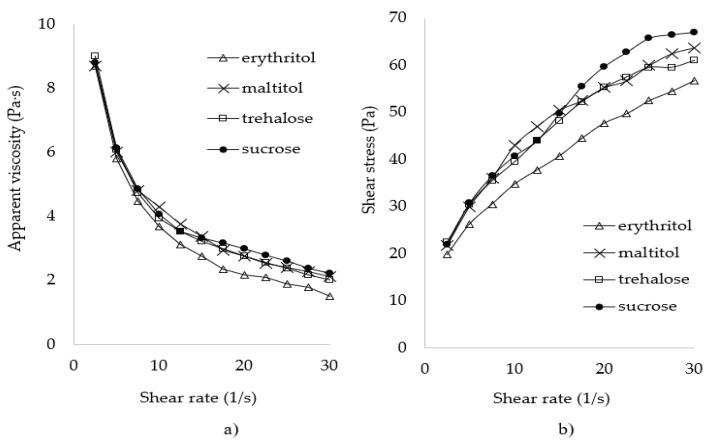
Rheological properties of the dough with sweetener: (**a**) viscosity curve; (**b**) flow curve.

The ingredients used to prepare the dough determine its rheological properties. Research reports indicate that it is important for the dough to have high viscosity because it slows down gas diffusion and enables gas retention at the early stage of the baking process [30]. As shown in Figure 1, regardless of the sweetener applied, the viscosity of the dough decreased as the shear rate increased (Figure 1a). The sweeteners did not affect the shape of the flow curves. Considering their rheological properties, the analyzed dough may be classified as a shear thinned fluid (Figure 1b).

The occurrence of the phenomenon of shear thinning may be explained by the process of dough structure damage. Shear thinning of a sample at an increasing rate results in damage to and the coalescence of air bubbles [30]. In addition, the rate of disruption of the existing intermolecular bonds is higher than the rate of their formation [31]. When the stress increases, the values of the shear force may exceed the values of the forces of the structure-forming interactions, thereby leading to material structure reorganization [32]. It consequently results in a reduction in the dough’s resistance to shearing, which is manifested in decreasing values of apparent viscosity. The low viscosity of the dough with erythritol may be explained by the easy coalescence or gas diffusion between bubbles [10]. The Ostwald–de Waele equation was used to compute the consistency index and the flow behavior index (Table 1).

The model’s fit to the experimental data was estimated based on the coefficient of determination (R^2^). In the Ostwald–de Waele power model, the values of the R^2^ coefficient ranged from 0.986 for the dough with sucrose to 0.999 for the dough with erythritol. The consistency index (K) of the dough with erythritol was about 33% lower than the K of the dough formed with the other sweeteners (Table 1). The flow behavior index (*n*) of the dough with trehalose (0.353) was similar to that of the dough with sucrose (0.379). Statistically significantly higher values of n were determined for the dough with maltitol (0.424) and erythritol (0.532) (Table 1). This means that dough with erythritol was the least viscous. Hao et al. [10] reported that the behavior of maltitol was closest to that of sucrose in a sponge cake system and that the molecular weight of sugar is the main factor determining the solution’s viscosity. Various authors have reported that disaccharides had a greater effect on the viscosity of protein solutions than monosaccharides with the same number of monosaccharide units [10,31,33]. The density of a dough is an essential physical property and related to the number of air bubbles incorporated into the dough [30], which depends on the aeration and mixing process and the dough’s ingredients. Proper management of key parameters such as time, aeration and mixing speed, dough temperature, water absorption of the flour, and amount of water in the dough can significantly improve the dough-making process and its impact on the rheology and density [34]. All the ingredients in the dough compete with each other for water, and these interactions produce different types of bonds, making the relative amount of ingredients a critical factor [35]. The density of the dough with maltitol was 0.437 g/cm^3^ and was the same as that of the dough with sucrose (0.430 g/cm^3^) (Table 1). The density of the dough with erythritol and trehalose was lower, about 21% and 41%, respectively (Table 1). This indicates that more air may be incorporated into the dough containing sucrose or maltitol than into the dough with trehalose or erythritol. A dough with a lower density, and thus with larger volumes of air incorporated into it, might be expected to produce sponge cakes with higher final volumes; however, there are other factors that influence the final volume, such as gas loss during processing, starch gelatinization, and possible collapse of the structure after baking [36].

The pH values of the dough with trehalose and maltitol were similar (7.63–7.65), whereas that of the dough with erythritol was higher (7.81) compared with the pH of the dough with sucrose (7.72) (Table 1). The pH values of dough with erythritol, maltitol, xylitol, and sucrose were also analyzed previously [10], revealing no effect of these sweeteners on the dough’s pH.

### 2.2. Sponge Cake Water Content, Water Activity, and Color

A drawback of sponge cake is the very short period during which they stay fresh, which results from their delicate and easily degradable structure and from their water content. Baked products made without preserving agents may be stored for only a few days [37]. The water content of the sponge cake with erythritol, maltitol, and trehalose was about 38.3 (g/100 g), and it was only slightly higher than that of the sponge cake with sucrose (Table 2). The water activity (a_w_) in the studied sponge cakes ranged from 0.878 to 0.935 (Table 2).

The high values of this parameter were indicative of the low microbiological stability and, consequently, of the short shelf life of these products. The a_w_ of the sponge cake with trehalose was the same as that of the cake with sucrose. The sponge cake with maltitol was characterized by the highest, and that with erythritol by the lowest, water activity. The erythritol reduced a_w_ much more than maltitol, most likely due to its lower molecular weight and higher hygroscopicity [38]. The a_w_ of the sponge cakes with sucrose was similar to that reported by Janjarasskul et al. [37]. The lowest a_w_, 0.860, was achieved by Hesso et al. [39]. However, these authors used different baking temperatures and durations.

**Figure 2:**
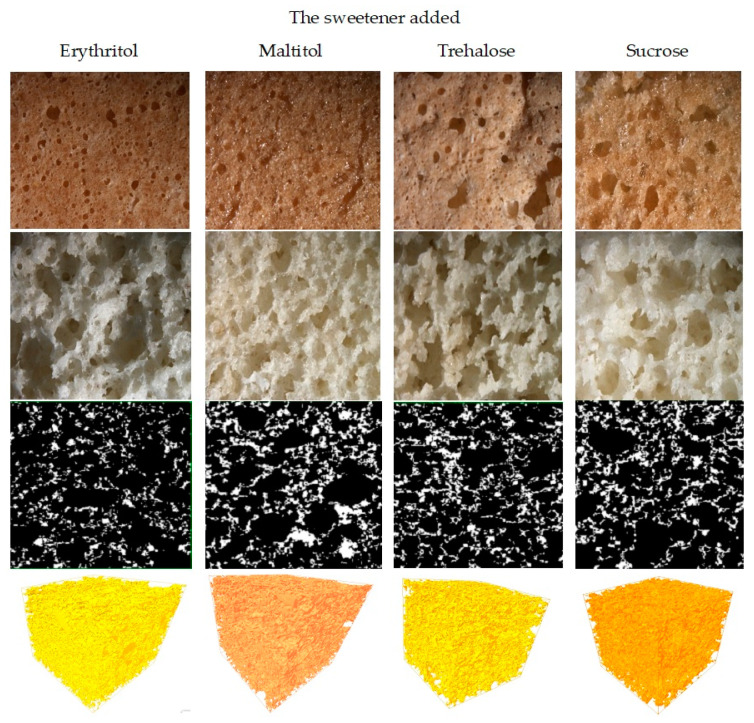
Images and characteristics of the porous structure of sponge cakes with sweetener.

The effect of sucrose substitution on the color of the crust and crumbs of sponge cake is presented in Table 2. In addition, photographs of crusts and crumbs of the studied sponge cakes are shown in Figure 2. The crust of the sponge cake with maltitol was shiny and similar to the crust of the control cake (with sucrose). In contrast, the color of the crust of the sponge cakes with trehalose and erythritol was more matt (Figure 2). The sweetener had a significant effect on the studied color parameters (L* (lightness), C* (chroma), and h_ab_* (hue)) (Table 2). Both the crust and crumbs of the sponge cakes with sweeteners were significantly lighter than those of the cakes with sucrose. As trehalose is not a reducing sugar, it does not undergo Maillard browning reactions. It does not caramelize [40]. Therefore, the sponge cake with trehalose had a lighter crust and crumbs and a less saturated color than the sponge cake with sucrose (Table 2). The lightest was, however, the sponge cake with erythritol, which was indicated by the highest value of its L* parameter. The chromaticity of the crust was lower, but the crumbs were characterized by a more saturated color than the sample with sucrose. Polyols do not participate in Maillard reactions in the presence of amino acids because of the lack of aldo and keto groups [15]. The use of polyols as replacing agents in sugar-free baking products frequently results in their having a lighter color [19]. All studied sweeteners had a significant effect on the hue of the sponge cake with sucrose (Table 2). The crust of the sponge cake had positive values of the h_ab_ parameter, which meant that its color was warm and ranged from yellow to orange. The highest value of the hue parameter was noted in the crust of the sponge cake with sucrose, whereas the lowest values were determined for both the crust and crumbs of the sponge cake with erythritol (Table 2). In turn, the highest value of the hue parameter of the crumbs was found for the sponge cake with maltitol. The negative values of this parameter indicate that the hue of the color of these crumbs was cold (similar to the green color).

### 2.3. Real Density, 2D Microstructure, and 3D Microstructure of Sponge Cake

The real density of the sponge cake with erythritol, maltitol, and trehalose was about 5% higher than that of the sponge cake with sucrose (Table 3). The replacement of sucrose reduces the stability of the air pores and leads to the cracking of the sponge cake’s walls. Consequently, during baking, it is difficult to stop the gas from diffusing [40]. The sucrose increases the starch gelatinization temperature and the protein denaturation temperature, and thereby delays these processes, which leads to air bubble expansion [4]. According to Ronda et al. [19], the lower volume and the higher density of the cake with xylitol may result from the incapability of polyols to increase the starch gelatinization temperature.

The sweeteners had a significant effect on the microstructure of the sponge cake (Figure 2). The crust of the sponge cake with erythritol had both large and small pores. The highest number of small pores with a round, regular shape was observed in the crust of the sponge cake with maltitol. This sweetener made the crust shiny, similar to that of the sponge cake with sucrose. The crust of the sponge cake with trehalose was characterized by large pores with irregular “jagged” shapes. A cake’s volume is related to the amount of air incorporated into the baked product [1].

**Figure 3:**
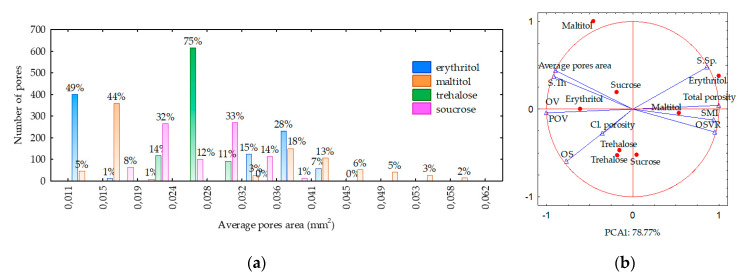
Structure of sponge cakes with sweetener: (**a**) distribution of pore size; and (**b**) PCA diagram of the 2D and 3D structural parameters. POV, percent object volume; OSVR, object surface/volume ratio; OV, object volume; OS, object surface; SMI, structure model index; S.Th, structural thickness; S.Sp, structure separation.

The crumbs of the sponge cake with erythritol and maltitol were characterized by a high number of small pores that were densely arranged compared with the crumbs of the sponge cake with sucrose (Figure 2). These observations were confirmed by the analysis of the 2D and 3D microstructures measured with microtomography. However, it has been found that cakes with similar volumes can be different in their pore size distribution (Figure 3a). The size of the pores ranged from 0.005 mm^2^ to 0.062 mm^2^ and was different for the sponge cakes with sweeteners and sucrose (Figure 3a). The size of the pores was higher in the sponge cake with maltitol. The distribution shows that the sponge cake with maltitol had the most diverse range of pore sizes; 49% of the pores were very small (<0.019 mm^2^), 21% of the pores were from 0.032 mm^2^ to 0.041 mm^2^, and 29% of the pores were large (between 0.041 mm^2^ and 0.062 mm^2^). The sponge cake with erythritol had a lot of small pores (49% of the pores were <0.015 mm^2^) and a lot of large pores (50% of the pores were between 0.032 mm^2^ and 0.045 mm^2^). The sponge cake with trehalose had the most homogeneous structure; 98% of the pores were between 0.020 mm^2^ and 0.030 mm^2^. On the other hand, 8% of the pores in the sponge cake with sucrose had an area <0.019 mm^2^, 44% of the pores had an area <0.028 mm^2^, and 47% of the pores had an area <0.036 mm^2^ (Figure 3a).

The average pore area of the sponge cake with maltitol was significantly larger than that of the other tested samples of sponge cake (Table 3). The structural thickness (S.Th) value, representing the wall thickness of the pores, was the highest in the sponge cake with maltitol (0.075 mm) (Table 3). In contrast, the other sweeteners tested resulted in the S.Th of 0.069 mm, which was lower than that of sponge cake with sucrose (0.071 mm). The sponge cakes with sweeteners had a structure separation (S.Sp) value ranging from 0.287 mm for sponge cake with trehalose to 0.354 mm for sponge cake with maltitol (Table 3). 

All of the SC samples were of open porosity (Table 3). Porous food materials with an open cell structure contain pores that are connected to each other through an interconnected network, which is soft compared with closed-pore foam structures. The closed pores are not interconnected, and they have a higher compressive strength due to their dense structure [1]. Multiple pores with different shapes, sizes, and 3D orientations have been found. The percent object volume (POV) in the sponge cakes was measured based on the micro-CT 3D images. The POV corresponds to the percentage of the voxel volume of the solid in relation to the total voxel volume.

According to Cafarelli et al. [29], the basic parameter characterizing the average complexity of the structure is the object area-to-volume ratio (OSVR). In addition, it is the basis for the model-dependent estimation of thickness, i.e., the mean size and distribution of pores occurring in each sample. The sponge cake with maltitol and trehalose had POV, OSVR, and total porosity values similar to those of the sponge cake with sucrose (Table 3).

In contrast, the sponge cake with erythritol differed, having the lowest POV (16.45), the highest OSVR (41.24), and the highest total porosity (83%). The sponge cake with sweeteners had significantly fewer closed pores compared with the control cake. The sponge cake with erythritol and maltitol had a structure model index (SMI) that was about 9% higher than that of the sample with sucrose. The anisotropy coefficient (DA) of sponge cakes with sweeteners was lower than that of the sponge cake with sucrose (14% for the sponge cake with maltitol and about 30% for the sponge cake with erythritol and trehalose, Table 3).

### 2.4. Sponge Cake Mechanical Properties 

The hardness of the sponge cakes with erythritol, maltitol, and trehalose was the same as that of the control cake (Table 3). Similarly, Psimouli and Oreopoulou [6] did not find differences in cake hardness when sucrose was replaced with maltitol. The hardness of the sponge cake is a result of multiple factors, including the starch gel, the gluten network, and the air phase fraction in the cake [10]. In our study, the structure of the sponge cakes with sweeteners was dissimilar. However, the total porosity was similar, and this could possibly affect the hardness. The sponge cake with erythritol had the highest porosity and its hardness was the lowest. The applied sweeteners caused statistically significant differences in the cohesiveness of the sponge cakes (Table 3). The highest value of this parameter (0.73) was found for the sponge cake with sucrose and the lowest value of this parameter (0.53) was found for the sponge cake with erythritol. These results indicate that less energy was required during the second compression. Cohesiveness is important because it affects consumer perceptions when chewing food. In addition, it refers to the rate at which food disintegrates under mechanical action and may also be defined as the resistance of food to traction [36]. The springiness is a measure of the ability of a cake to recover after compression. Low springiness was found for the sponge cakes with erythritol and trehalose (0.79 and 0.89, respectively; Table 3). The springiness of the sponge cakes with maltitol (0.94) was the same as that of the sponge cake with sucrose. Other authors have reported a decrease in springiness with the addition of an emulsifier. The use of emulsifiers is known to reduce the strength of interactions between starch and protein fractions of flour in bakery systems and can lead to a rather crumbly product [14,30]. Rodríguez-García et al. [30] found that the decrease in springiness was associated with a decrease in the volume and in the total percentage of cells and, therefore, with a denser crumb structure. The sponge cake with erythritol and trehalose had the lowest water activity, and this may have contributed to the low springiness. Moreover, these sponge cakes had pores with a lower structural thickness (S.Th) and a higher OSVR than the sponge cake with maltitol and sucrose. The mechanical behavior is due to the cellular structural features of the cakes; more precisely, the cell wall size distribution [27]. Adhesiveness is essential in consumer evaluation. The higher the value is above zero, the better it is. It is defined as the work necessary to overcome the attractive forces between the product and a specific surface and depends on the cohesive forces in the product as well as on its viscous properties. The adhesiveness of the sponge cakes with maltitol and erythritol was the same as that of the sponge cake with sucrose, whereas significantly higher adhesiveness was found in the samples with trehalose (Table 3).

### 2.5. Sponge Cake Sensory Properties

**Figure 4:**
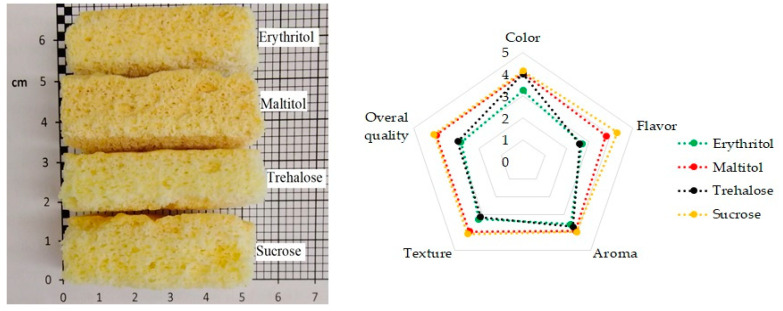
Sensory properties of sponge cakes with sweetener.

All panelists participating in the consumer test stated that they eat sponge cake. Among the respondents, the largest group (58%) comprised consumers who reported that they eat sponge cakes less than once a month; 30% of the respondents answered that they eat sponge cakes several times a month. Only 12% reported eating sponge cakes 1–2 times a week. None of the respondents reported that they eat this type of product every day. The sensory properties are shown in Figure 4. It was found that 100% replacement of sucrose allows for a porous structure to be obtained. Polyols and trehalose as sucrose replacers can improve the nutritional value of cakes. In addition, polyols can reduce the caloric value of cakes [11,14].

According to the consumers, sponge cakes with sucrose had the best flavor, texture, and overall quality. The consumers also liked the sponge cakes with maltitol (Figure 4). The sweetness of this substance ranges from 0.8 to 0.9 in relation to sucrose. Ghosh and Sutha [15] reported that products in which sucrose was replaced with maltitol had similar properties. The use of erythritol and trehalose resulted in a worse flavor (Figure 4). The sponge cake with maltitol rose the most (Figure 4). An analysis of its structure showed that its pores had the largest area. This contributed to a better opinion of these samples among the panelists. It has been proven that a higher number of pores with a larger exposed surface area allows for an intense taste to access the mouth during mastication. This means that it is the larger surface area in contact with the tongue or other sensory organ that provides the intense taste of the product [41].

An effect of the sweetener on the aroma was not detected. The colors of the sponge cakes with maltitol and trehalose were similar and acceptable. The sponge cake with erythritol had the least-desirable, lighter color. None of the tested sweeteners had reducing properties. Sucrose acts as a crystallizing and hardening agent in biscuits, giving the final product a brown surface that is acceptable to consumers. Similar properties may be shown by trehalose and maltitol [10].

### 2.6. Correlations between Structural, Mechanical, and Sensory Properties

**Figure 5:**
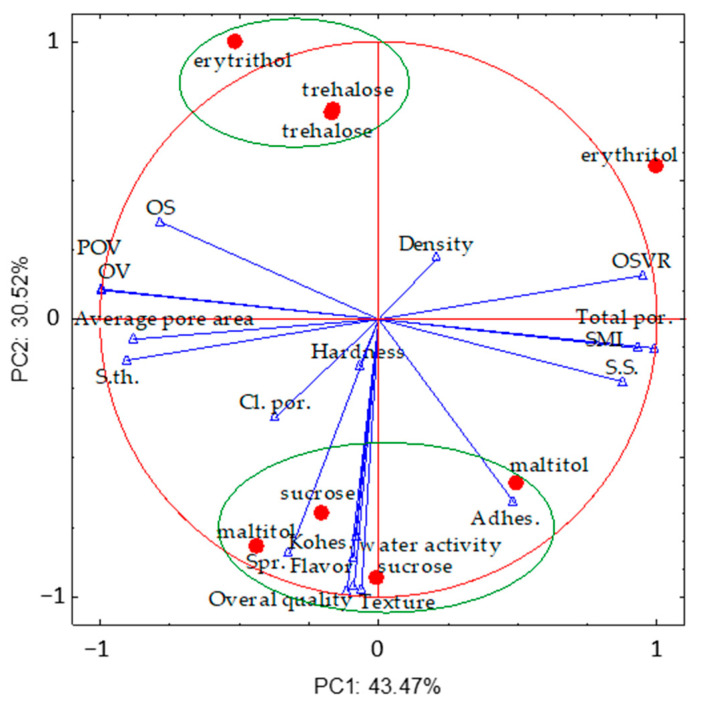
PCA diagram. Structural parameters: Cl. por., closed porosity; Total por., total porosity; POV, percent object volume; OSVR, object surface/volume ratio; OV, object volume; OS, object surface; SMI, structure model index; S.Th, structural thickness; S.Sp, structure separation. Mechanical parameters: Hardness; Cohes., cohesiveness; Adhes., adhesiveness; Spr., springiness. Sensory parameters: Texture; Overall quality; Flavor.

Principal component analysis (PCA) was applied to identify correlations between the structural, mechanical, and sensory parameters of the sponge cakes with sweeteners. PCA provides a visual representation of the correlations between parameters and samples and provides insights into how measured parameters cause some samples to be similar or different from each other (Figure 5). The first two principal components (PCs) were able to explain 74% of the variance in all measured parameters. The PCA revealed that the PC1 and PC2 components explained 43.5% and 30.5% of the total variance, respectively. PC1 was formed by structural parameters and PC2 by mechanical and sensory parameters. The samples with sucrose were more or less grouped according to the sweetener in the space of the first two PCs. The samples with maltitol and trehalose had PC2, as did the samples with sucrose. Only in the sponge cake samples with erythritol was PC1 significantly different. The identified correlation coefficients were significant at *p* < 0.05. The analysis of correlations between the investigated parameters demonstrated that only closed porosity was strongly correlated with the hardness (*r* = 0.787) of the sponge cakes. The structure separation and object surface were strongly correlated with adhesiveness (*r* = 0.754 and *r* = −0.821, respectively). The texture-assessed sensory effects were correlated with cohesiveness (*r* = 0.814) and springiness (*r* = 0.775). Overall quality was correlated with springiness (*r* = 0.875) and water activity (0.788). The structural and mechanical properties of food influence the perception of texture, which contributes to food quality and the pleasure of eating, and also influence digestion and lipolysis, which contribute to nutrition and health [24]. However, in our study, no correlation was found between the structural parameters and the sensory parameters.

## 3. Materials and Methods

### 3.1. Dough and Sponge Cake Formulation

The experimental material included dough and sponge cakes. The sponge cakes were prepared under laboratory conditions according to the formulas in which the percentage content of individual ingredients was the same, but the sweetener differed. Sucrose (sugar powder, DIAMANT, Glinojeck, Poland) was replaced with erythritol (Brenntag, Poland Sp. Z o.o.) or maltitol (Brenntag Poland Sp. Z o.o., Warsaw) or trehalose (Hortimex Sp. Z o.o. Konin, Poland). All sweeteners were ground to the same fraction size of 5–10 μm. Wheat flour was 450-type (with a water content of 13%) (Polskie Młyny, Teresin, Poland), potato starch was from Hortimex (Sp. Z o.o., Konin, Poland), and fresh eggs were from a farm at the Warsaw University of Life Sciences (Warsaw, Poland).

The formulation of sponge cake used in this study was: fresh egg white (150 g), sweetener (100 g), fresh egg yolk (75 g), wheat flour (72 g), and potato starch (25 g).

Ingredients were weighed on a laboratory balance (Radwag, Radom, Poland) exactly to 0.1 g. The doughs containing erythritol, maltitol, trehalose, and sucrose were prepared as follows. Egg white was whipped for 2 min with a mixer (Zelmer, Rzeszów, Poland) at the highest revolution speed. Then, the mixer speed was decreased to a medium level, and then the sweetener was added in two batches to the whipped froth at 1-min intervals. Then, egg yolks were added in two batches at 2-min intervals. Next, sifted wheat flour and potato starch were added to the dough, which was then carefully mixed with a whisk. The total time of whipping and mixing was 8 min.

The dough with different sweeteners was baked on an aluminum tray at 210 °C for 10 min. After baking, pieces (size: 65 × 50 mm) were cut out from the baked sponge cakes and cooled for 30 min before further analysis.

### 3.2. Rheological Properties, Density, and pH of the Dough

Rheological measurements were carried out with a Brookfield DV-III V3.3 RV rotary viscometer (U.S.A.) at 22 °C in the shear rate range of 1–30 s^−1^ using an RV-29 mandrel. 

Quantities of 13 mL of dough were subjected to shearing. The rheological measurements were made within 15 min of the preparation of the dough [6]. The measurement time for one sample was 120 s. The following rheological model was applied for the flow curve description: ${\tau =K\gamma }^{n}$, where: τ is the shear stress (Pa), γ is the shear rate (s^−1^), K is the consistency coefficient (Pa·s^n^), and *n* is the flow behavior index. The parameters used as criteria to select the optimal model included the coefficient of determination R^2^.

The density was determined based on the mass and volume of the dough sample. The dough was put into a dry measuring vessel and weighed to an accuracy of 0.01 g. Measurements were performed in triplicate.

The pH value of the dough was measured using a pH meter with an IJ-44C glass electrode (Elmetron, Poland) at a temperature of 25 ± 1 °C.

### 3.3. Sponge Cake Water Content, Water Activity, and Color

The water content in crumbs was determined with the drying method (at 105 °C for 3 h) using samples (1 g) cut from the center of three cakes.

Water activity (a_w_) was determined using an AquaLAB device (Decagon Devices Inc., Pullman, WA, USA) at a temperature of 25 ± 1 °C. Determinations were performed in triplicate.

The color of sponge cakes was evaluated in the CIELab system using a Konica Minolta CM-5 colorimeter at the standard lighting of D65. Ten sponge cakes were measured at the bottom of the center for: L*—lightness (black/white), a*—redness (green/red), and b*—yellowness (blue/yellow).

Results obtained were used to calculate the color chroma C* = ((a*)^2^ + (b*)^2^))^0.5^ and the hue angle h_ab_ = arctan(b*/a*), where: C* = chroma; a* = contribution of red color; b* = contribution of yellow color; and h_ab_ = hue.

### 3.4. Real Density and Microstructure of Sponge Cakes

The real density of sponge cakes was determined using a helium stereopycnometer (Quantachrome Instruments, Boynton Beach, FL, USA). Measurements were performed on sponge cake samples weighing ca. 8 g.

The images of the sponge cake’s structure were taken using a Nikon SMZ1500 stereomicroscope equipped with a DS-Fi1 camera at 7.5× magnification.

The microstructure of the sponge cake was measured using a Skyscan 1272 X-ray micro-computed tomography system (v.1.1.7, Bruker Corp., Kontich, Belgium). The X-ray source was operated at a voltage of 40 kV and a current of 222 μA. Images were obtained with a rotation step of 0.4 over an interval of 0–360 and a two-frame average. Two samples were scanned for each type of sponge cake. The scanning time of one sample was 30 min. About 820 images were obtained and processed with the reconstruction software NRecon v. 1.7.3 (Bruker Corp., Billerica, MA, USA) to obtain 2D cross-sectional images (1344 × 896 pixels).

The images were reconstructed and analyzed using CTAn software v. 1.17 (Bruker Corp., Belgium). The following microstructural parameters were obtained: percent object volume (POV), object surface/volume ratio (OSVR), object volume (OV), object surface (OS), structure model index (SMI), anisotropy coefficient (DA), closed porosity, total porosity, the average size of pores, structural thickness (S.Th), and structure separation (S.Sp).

### 3.5. Sponge Cake Mechanical Properties

Texture profile analysis (TPA) was conducted in a TA.HD.plus Texture Analyzer (Stable Micro System, Godalming, UK). Samples of sponge cakes (20 × 32 × 10 mm) were subjected to a double compression test using an SMS P100 head. The sample was then compressed twice to 50% of the original height at the compression rate of 1 mm/s. The first compression cycle was 25%. When the compression stroke was completed, the plunger remained at rest for 5 s, after which it compressed the same sample by 25%.

The test was conducted on ten replicates of each cake formulation. Hardness was the peak force measured during the first compression cycle. Cohesiveness was the ratio between the positive areas of the second cycle and the first cycle and is dimensionless. Springiness was the ratio between the time difference of the second cycle and the first cycle and is dimensionless.

### 3.6. Sensory Properties

Sensory testing was carried out by 40 semi-trained panelists (students) of the Faculty of Food Sciences (32 women and 8 men, all between 18 and 25 years of age). The panelists provided a description of all sensory parameters with an evaluation sheet. In the first stage, the consumers′ task was to fill in a questionnaire regarding their consumption of sponge cake. The consumers received four coded samples of sponge cake with various sweeteners; they were instructed to evaluate five parameters (color, flavor, taste, texture, and overall quality) of the sponge cakes. We used the following categories of sensory analysis on a hedonic scale from 1 to 5: 1—very bad, 2—bad, 3—acceptable, 4—good, 5—very good. Water (room temperature) was used as a neutralizer between the evaluation of different samples. The important characteristics determining the quality and attractiveness of sponge cake were selected using the method of random sampling.

### 3.7. Statistical Analysis

An analysis of variance (ANOVA) was conducted using Statistica 12 PL and the significant differences between mean values were determined using Duncan′s multiple range test at a significance level of *p* < 0.05. In the case of an abnormal distribution, division into homogeneous groups was performed using the nonparametric multiple comparison test (the Kruskal–Wallis test). Principal component analysis (PCA) was performed as well.

## 4. Conclusions

The replacement of sucrose by erythritol, maltitol, and trehalose resulted in the development of individual rheological properties of the dough and structural, mechanical, and sensory properties of the sponge cake. The dough with erythritol was characterized by the lowest consistency index (i.e., it was the least viscous). Furthermore, the consistency index of the dough with maltitol and trehalose was similar to that of the samples with sucrose. The crust and crumbs of the sponge cakes with sweeteners were lighter than those of the sponge cake with sucrose. The sponge cake with maltitol had the broadest pore size distribution, followed by the sponge cakes with erythritol, sucrose, and trehalose. In turn, the sponge cake with maltitol had relatively thicker pore walls. The hardness, springiness, cohesiveness, and sensory qualities of the sponge cake with maltitol were similar to those of the sponge cake with sucrose. The sponge cake with trehalose was characterized by very high adhesiveness and low sensory quality. Substitution of the commonly used sucrose with erythritol and trehalose allowed for sponge cakes with a porous structure to be prepared, but the flavor was not acceptable. On account of the sensory properties of the sponge cakes, we recommend the use of maltitol to replace sucrose.

Although no significant correlation was found between the sensory parameters and the structure, the higher sensory quality of the maltitol sponge cake was due to its taste and could be due to the presence of larger pores than in the other samples tested.

Among the mechanical parameters, hardness was correlated to closed porosity (*r* = 0.787) and adhesiveness was correlated to structure separation (*r* = 0.754) and pore surface area (*r* = −0.821). Sponge cakes with sweeteners in the place of sugar may be attractive to consumers. The application of sweeteners in sponge cakes represents one way to reduce the energy value of a product with a high market share. The individual traits of these baked products may be advantageous to customers not only due to the reduced energy value but also to their structure. The significantly lighter and less intense colors of sponge cakes with sweeteners may be modified through the use of natural pigments, which could additionally increase the nutritive value of these products.

## Figures and Tables

**Table 1:** Rheological parameters, density, and pH value of dough with sweetener (each value is presented as mean ± SD; values with the same letter in a column are not significantly different at a *p* < 0.05).

The Sweetener Added	Consistency IndexK (mPa·s^n^)	Flow Behavior InDex *n* (-)	R^2^	Density of Dough(g/cm^3^)	pH
Erythritol	10.4 ± 0.7 ^a^	0.532 ± 0.082 ^c^	0.999 ± 0.001	0.252 ± 0.005 ^a^	7.81 ± 0.04 ^b^
Maltitol	15.9 ± 1.1 ^b^	0.424 ± 0.010 ^b^	0.988 ± 0.006	0.437 ± 0.006 ^c^	7.65 ± 0.04 ^ab^
Trehalose	15.2 ± 0.4 ^b^	0.353 ± 0.086 ^a^	0.980 ± 0.021	0.313 ± 0.008 ^b^	7.63 ± 0.01 ^a^
Sucrose	14.0 ± 0.5 ^b^	0.379 ± 0.119 ^a^	0.964 ± 0.042	0.430 ± 0.010 ^c^	7.72 ± 0.04 ^ab^

**Table 2:** Physicochemical parameters of sponge cakes with sweetener (each value is presented as mean ± SD; values with the same letter in a column are not significantly different at a *p* < 0.05 level).

The Sweetener Added	Moisture (g/100 g)	Water Activity (a_w_)	Crust			Crumb		
			L*	C*	h_ab_	L*	C*	h_ab_
Erythritol	38.3 ± 2.3 ^a^	0.878 ± 0.061 ^a^	71.22 ± 0.05 ^d^	31.35 ± 0.03 ^b^	1.31 ± 0.00 ^a^	82.45 ± 0.05 ^d^	23.59 ± 0.11 ^d^	−1.47 ± 0.00 ^a^
Maltitol	38.5 ± 0.2 ^a^	0.935 ± 0.028 ^c^	67.47 ± 0.15 ^a^	28.68 ± 0.34 ^a^	1.38 ± 0.00 ^c^	82.05 ± 0.11 ^c^	23.02 ± 0.06 ^b^	−1.42 ± 0.00 ^d^
Trehalose	38.3 ± 1.0 ^a^	0.897 ± 0.028 ^a^	70.45 ± 0.10 ^c^	33.83 ± 0.06 ^c^	1.34 ± 0.00 ^b^	78.84 ± 0.07 ^b^	23.31 ± 0.04 ^c^	−1.43 ± 0.00 ^c^
Sucrose	37.0 ± 2.4 ^a^	0.902 ± 0.030 ^b^	68.00 ± 0.09 ^b^	36.77 ± 0.18 ^d^	1.43 ± 0.00 ^d^	78.48 ± 0.08 ^a^	22.80 ± 0.11 ^a^	−1.45 ± 0.00 ^b^

**Table 3:** The 2D and 3D structural and textural parameters of sponge cakes with sweetener (each value is presented as mean ± SD; values with the same letter in a line are not significantly different at a *p* < 0.05).

Structural and Textural Parameters	The Sweetener Added			
	Erythritol	Maltitol	Trehalose	Sucrose
Real density (g/cm^3^)	1.595 ± 0.120 ^b^	1.628 ± 0.027 ^b^	1.574 ± 0.151 ^b^	1.502 ± 0.066 ^a^
Average pores area (mm^2^)	0.025 ± 0.002 ^a^	0.030 ± 0.014 ^b^	0.025 ± 0.002 ^a^	0.026 ± 0.002 ^a^
S.Th (mm)	0.069 ± 0.010 ^a^	0.075 ± 0.010 ^c^	0.069 ± 0.002 ^a^	0.071 ± 0.004 ^b^
S.Sp (mm)	0.351 ± 0.061 ^a^	0.353 ± 0.010 ^a^	0.287 ± 0.002 ^a^	0.316 ± 0.003 ^a^
Total porosity (%)	83.56 ± 7.23 ^a^	82.83 ± 4.13 ^b^	81.26 ± 0.28 ^b^	82.22 ± 082 ^b^
Closed porosity (%)	0.025 ± 0.025 ^b^	0.025 ± 0.012 ^b^	0.020 ± 0.002 ^a^	0.049 ± 0.047 ^c^
POV (%)	16.45 ± 7.23 ^a^	17.17 ± 4.13 ^b^	18.74 ± 0.28 ^b^	17.78 ± 0.82 ^b^
OSVR (1/mm)	41.24 ± 10.34 ^a^	37.79 ± 8.55 ^b^	39.69 ± 0.35 ^b^	38.51 ± 1.50 ^b^
SMI (-)	1.74 ± 0.79 ^b^	1.69 ± 0.65 ^b^	1.45 ± 0.04 ^a^	1.54 ± 0.50 ^a^
DA (-)	0.160 ± 0.049 ^a^	0.178 ± 0.081 ^a^	0.158 ± 0.005 ^a^	0.223 ± 0.004 ^b^
Hardness (N)	7.72 ± 3.41 ^a^	7.83 ± 1.85 ^a^	8.38 ± 1.6 ^a^	8.54 ± 3.36 ^a^
Cohesiveness (-)	0.53 ± 0.04 ^a^	0.69 ± 0.01 ^bc^	0.64 ± 0.03 ^b^	0.73 ± 0.02 ^c^
Springiness (mm)	0.79 ± 0.04 ^a^	0.94 ± 0.03 ^c^	0.89 ± 0.04 ^b^	0.94 ± 0.04 ^c^
Adhesiveness (g)	−0.49 ± 0.38 ^ab^	−0.24 ± 0.13 ^a^	−1.32 ± 1.06 ^b^	−0.29 ± 0.15 ^a^

S.Th, structural thickness (mm); S.Sp, structure separation (mm); POV, percent object volume; OSVR, object surface/volume ratio; SMI, structure model index; DA, degree of anisotropy (-).

## Abbreviations

a_w_	Water activity
τ	Shear stress (Pa)
γ	Shear rate (s^−1^)
K	Consistency coefficient (Pa·s^n^)
N	Flow behavior index
R^2^	determination coefficient
L*	Lightness
a*	Redness
b*	Yellowness
*C**	Chroma
*h_ab_*	Hue
POV	Percent object volume
OSVR	Object surface/volume ratio
OV	Object volume
OS	Object surface
SMI	Structure model index
DA	Anisotropy coefficient
S.Th	Structure thickness
S.Sp	Structure separation
R	Correlation coefficient
